# Age Classification in Forensic Medicine Using Machine Learning Techniques

**DOI:** 10.17691/stm2022.14.1.02

**Published:** 2022-01-28

**Authors:** G.V. Zolotenkova, A.I. Rogachev, Y.I. Pigolkin, I.S. Edelev, V.N. Borshchevskaya, R. Cameriere

**Affiliations:** Professor, Department of Forensic Medicine, First Moscow State Medical University (Sechenov University), 8/2 Malaya Trubetskaya St., Moscow, 119991, Russia; Researcher, Center for Information Technologies in Engineering of the Russian Academy of Sciences, 7а Marshala Biryuzova St., Moscow Region, Odintsovo, 143003, Russia; PhD Student, Big Data and Information Retrieval School, Faculty of Computer Science; HSE University, 11 Pokrovsky Boulevard, Moscow, 109028, Russia; Researcher, Center for Information Technologies in Engineering of the Russian Academy of Sciences, 7а Marshala Biryuzova St., Moscow Region, Odintsovo, 143003, Russia; Professor, Corresponding Member of the Russian Academy of Sciences, Head of the Department of Forensic Medicine, First Moscow State Medical University (Sechenov University), 8/2 Malaya Trubetskaya St., Moscow, 119991, Russia; Researcher, Center for Information Technologies in Engineering of the Russian Academy of Sciences, 7а Marshala Biryuzova St., Moscow Region, Odintsovo, 143003, Russia; Assistant, Department of Forensic Medicine; Privolzhsky Research Medical University, 10/1 Minin and Pozharsky Square, Nizhny Novgorod, 603005, Russia;; Assistant, Department of Forensic Medicine; Stavropol State Medical University, 310 Mira St., Stavropol, 355017, Russia;; Professor, AgEstimation Project, Institute of Legal Medicine, University of Macerata, Macerata, 62100, Italy

**Keywords:** forensic medicine, age diagnostics, age groups, machine learning techniques, nonlinear dimensionality reduction methods

## Abstract

**The aim of the study** was to assess the capabilities of age determination (age group) at death using classification techniques by histomorphometric characteristics of osseous and cartilaginous tissue aging.

**Materials and Methods:**

The study material was a database containing the findings of morphometric researches of osseous and cartilaginous tissue histologic specimens from 294 categorized male corpses aged 10–93 years. For data analysis and classification we used modern machine learning methods: k-NN, SVM, logistic regression, CatBoost, SGD, naive Bayes, random forest, nonlinear dimensionality reduction methods (t-SNE and uMAP), and recursive feature elimination for feature selection.

**Results:**

The used techniques (algorithms) provided effective representation of a complex data set (76 histomorphometric features), allowing to reveal the cluster structure inside the low dimensional feature space, thus fitting the classifier becomes even more reasonable. During feature selection, we estimated their importance for age group classification and studied the relationship between classification quality and the number of features inside the feature space. Data pre-processing made it possible to get rid of noise and keep most informative features, thereby accelerating a learning process and improving the classification quality. Data projection showed more well-defined cluster structure in the space of selected features. The accuracy of establishing certain groups was equal to 90%. It proves high efficiency of machine learning techniques used for forensic age diagnostics based on histomorphometric findings.

## Introduction

Age diagnostics is a key element in the identification of personality [1, 2]. Morphological changes of tissues and organs occurring in postnatal ontogenesis are not always the effects of ageing. The effect of a variety of factors including both endogenous (genetic predisposition, comorbidities, mass-height characteristics, and others), and exogenous (occupation, bad habits, ecological problems) causes the discrepancy between biological and real age with maximum bias of a resulting assessment in middle and elderly age groups [3, 4]. Linear regression used in expert practice contributes to the end result error increase in age prognosis. Considering the fact that tissue and organ aging processes have complex dynamics and cannot be described by simple linear dependencies, most researchers concur on inexpedience of such approach, since it fails to solve an objective [5–7].

Currently, the data set of quantitative aging indices of various organs and tissues has been stored [7–14]. For the most part, the established databases are “noisy”, since they contain a large number of heterogeneous indices hampering their processing and making a final solution. In such cases, it is reasonable to use nonlinear data mining with well-marked roundup properties [15]. Modern information technologies (machine learning techniques) are promising [16–18].

**The aim of the study** was to assess the capabilities of age determination (age group) at death using classification techniques by histomorphometric characteristics of bone and cartilaginous tissue aging.

## Materials and Methods

To attain the goal, the following study design was suggested:

1. feature selection and reduction of feature space using the chosen algorithm;
2. comparative analysis and classifier selection providing the maximum accuracy making the age prediction of an unknown person;
3. defining an optimal age interval to achieve the best performance in terms of maximum accuracy and reliability.

Forensic medical examination of an unknown person frequently states possible age indicating confidential or expected intervals, this referring is to a synthesizing assessment of numerous parameters to determine the boundaries of an age group the identified persons belongs to. This is an example of the classification task, which considers a variety of previously labeled objects described in some feature space and is used as a training sample to fit a model, which is capable to classify unlabeled data. To solve the task, we built the model classifying objects of similar nature. As a class label, we used an age group of an object. In the present study, for training and validation of different classifiers, we used databases of age-related changes of bone and cartilaginous tissues from 294 male corpses with known race (ethnic homogeneity) and age (from 10 to 93 years). Numerical values were taken from literature [2, 8–10] in micro-osteometric study of histological specimens of diaphysis and epiphysis of long bones (В1–В24) and thyroid cartilage (С1–С28).

On the basis of a default age group labeling recommended by VII All-Union scientific conference on age morphology, physiology and biochemistry, 7 age groups were distinguished: under 12 years; from 13 to 18 years; from 19 to 21 years; from 22 to 35 years; from 36 to 60 years; from 61 to 75 years; over 75 years. When forming the groups, we took into consideration literature data and the results of our previous studies. So, the ground for establishing the upper value of the second age group was the presence of epiphyseal plate, which can be found only in humans under 18 years of age.

For differentiated study, we divided the material into 10-year age intervals (Table 1).

**Table 1:** Distribution of autopsy objects (corpses) to study by ten-year intervals

Feature	Ten-year age intervals									
	1st	2nd	3rd	4th	5th	6th	7th	8th	9th	Total
Age (years)	≤10	11–20	21–30	31–40	41–50	51–60	61–70	71–80	≥81	
Number of observations	10	41	50	30	35	35	34	32	27	294

In order to fit a model to classify an age group of people using the given features, the following machine learning algorithms were used: random forest, CatBoost, k-NN, logistic regression, SGD, SVM, naive Bayes, t-SNE and uMAP, Python programming language, scikit-learn library. Within the study, we applied the techniques based on conceptually different approaches. Their brief summary is as follows.

*k-nearest neighbors algorithm (k-NN)* keeps the information on all objects of a training sample. For a new object, which is going to be classified, the closest points in the data of distance metric given in advance are found. Among the nearest k-points, the most common class is determined, which will be used as a model output.

In case of a classical logistic regression, we used the following methods: logistic regression from sklearn library and stochastic gradient descent (SGD) from Vowpal Wabbit based on a gradient descent — an iterative process, during which model weights are updated.

*Support vector machine (SVM)* in contrast to logistic regression, which has prerequisites, is based on object set geometry in feature space. SVM constructs maximum-margin hyperplane between objects of different classes, and it can be constructed both in an initial feature space and also in its new representation resulting from using a kernel — the function, which makes points in other feature space according to initial data. Thus, a separating hyperplane in the initial feature space differs from that in the initial space.

*Naive Bayes classifier* arranges the objects based on Bayes’ theorem. The approach utilizes even a small amount of data available for learning, evaluation of parameters, and classification.

*Decision tree* models a decision-making process by an expert. The model has a graph structure, in each tree node there is a decision rule that defines the next node, and in the tree leaves, there are the resulting labels of classes. Tree traverse from the roots and further according to the rules and feature values in a certain object defines the classification procedure.

*Random forest* is an example of an ensemble of decision trees. Simultaneously, several trees are fitted using different subsets of features during a fitting process. A final result is obtained by voting: each tree provides a class label, and the one receiving the majority vote in the ensemble is used as a final result.

*CatBoost* is based on a gradient boosting, in contrast to random forest, which is an example of bagging demonstrating an alternative way of model assembling. The essence of boosting usage is in the combination of weak (with low generalization ability) functions (low depth trees were used within the present study), which are fitted during an iterative process when at every step a new model is learnt using the data on the errors of the previous ones.

Classification quality was evaluated during cross-validation. The experiments were carried out on 5 folds. The quality of classification algorithms was estimated by F1-score metrics, and a model with the highest parameter value was chosen. To assess the classification, an error matrix was plotted which enabled to comprehend how a model was making errors, and where misclassified objects were referred to; as well as distinguish classes, the work with which causes most errors. This information was used to search ten-year age intervals in each class during the experiments, which can be achieved if there is an optimum relationship between quality of classifier work and intraclass age dispersion. We analyzed ROC curves plotted for each class: diagram form and area enabled to reveal problem classes (age groups).

## Results

**Figure 1:**
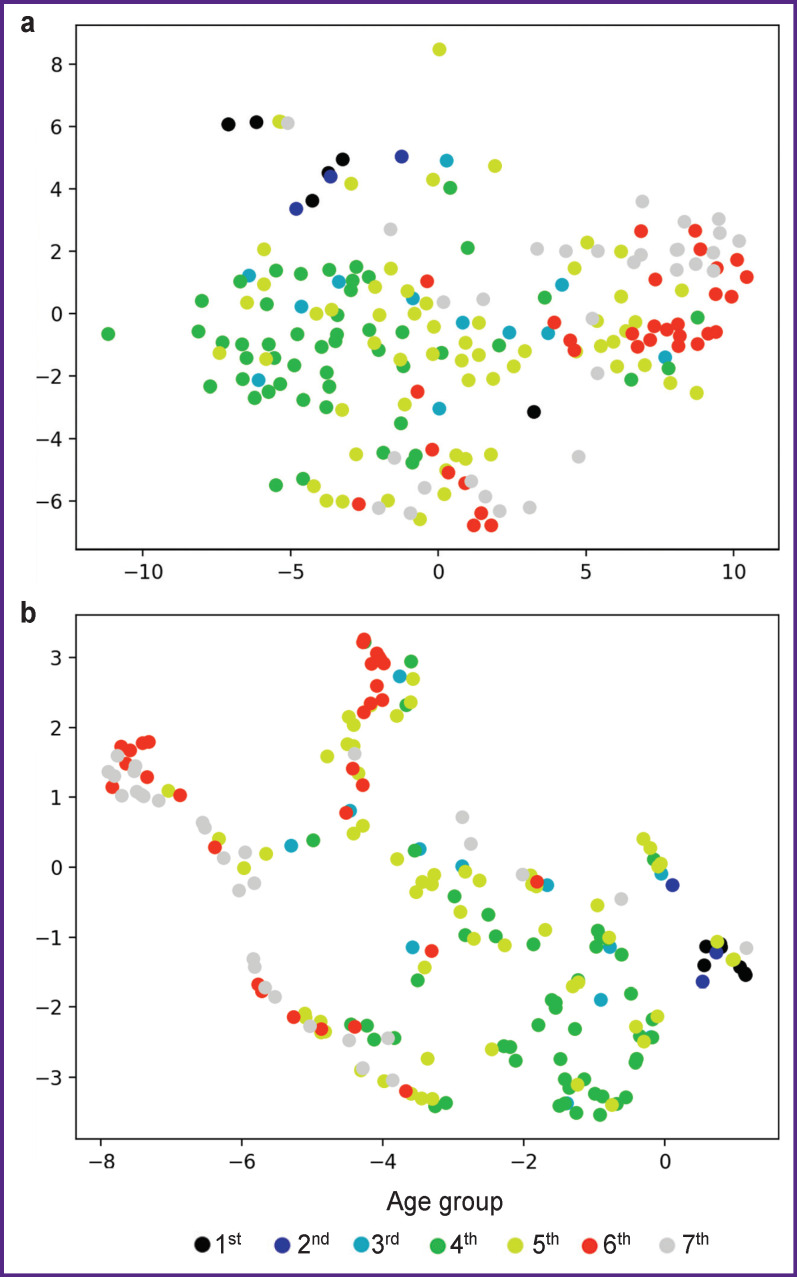
Two-dimensional display of initial data before feature selection: (а) using t-SNE; (b) using uMAP

Initial data was presented in the form of two-dimension images taken with the use of nonlinear dimension reduction techniques: t-SNE and uMAP enable to reduce dimension of feature space for the following data imaging. The techniques enable to get the low-dimensional representation of objects in such a way that the objects similar in the initial feature space are modeled by closely adjacent points, while dissimilar points are located as far apart as possible. Figure 1 represents the imaging results indicating close intermingling of age groups without evident clustering before feature selection. Those from age groups 1 (under 12 years) and 2 (13–18 years), which at this stage are visually separable, stand apart.

**Figure 2:**
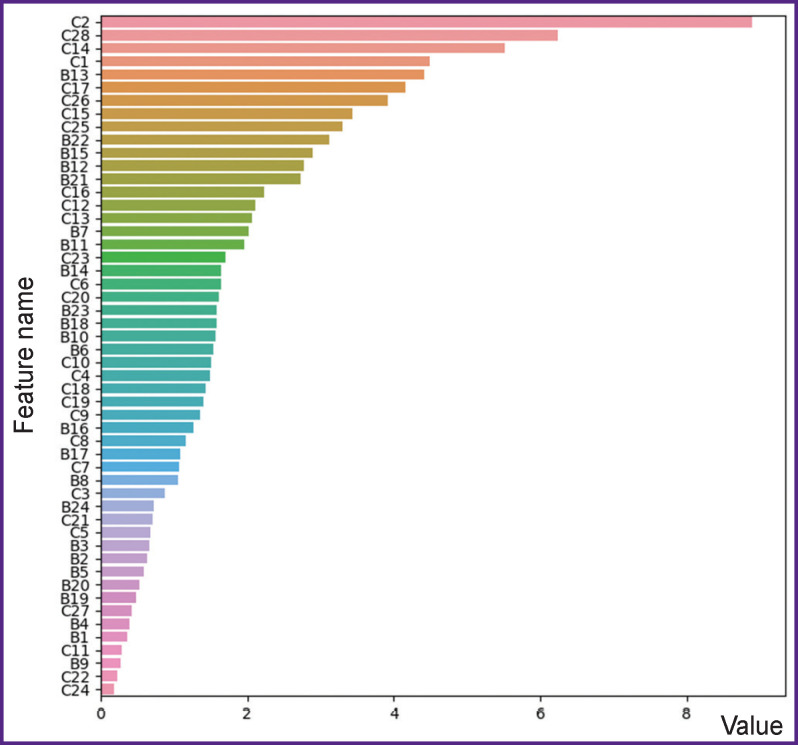
Informativity of features [2, 8–10]

The following stage was feature selection using recursive feature elimination involving decision trees and Gini coefficient (Figure 2). During the procedure, a model based on the selected algorithm was initially trained on all features, among which the less informative ones were chosen. In our case, importance is considered as a feature contribution to Gini coefficient value decrease when searching optimal split, i.e. the way, how efficiently we can separate the objects of different classes using a certain feature. This feature is eliminated, and the described procedure is repeated, the informative capacities of features are recalculated.

According to the findings, quantitative characteristics of age-related changes in the thyroid cartilage make the major contribution to target clustering; the changes relate to the processes of maturation and further ossification of the cartilaginous tissue itself, the replacement of reticular tissue by adipose tissue. Figure 2 shows the ranked list, where the following characteristics have the leading positions: osseous tissue area per field of vision of a histological specimen of the thyroid cartilage (С2), weighting (С28) in the thyroid cartilage radiograph of osseous (С1) and cartilaginous (С17) tissues. In addition, one should consider both cartilaginous tissue area and new cartilage zone thickness (С23), and also its correlation with the mature cartilage zone (С25). The areas of adipose (С14) and reticular (С15) tissues per field of vision of the thyroid cartilage also characterize the cartilage ageing and atrophic process in it. It stands to mention the validity of selecting not just osseous tissue area, but considering dimensional characteristics of trabecula per field of vision: their thickness (С13) and area (С6).

An expert and comparative analysis confirmed the practicability of selecting microosteometric indices as objective markers of age-related osseous tissue changes. The significance of characteristics indicating morphological changes in histostructure was noted, primarily, in the diaphysis compact bone: the thickness of inner (В10) and outer (В12) circumferential lamellae, osteone area (В11), and their quantitative characteristic (В23). Such remodeling parameters as Haversian canal diameter (B21) and its relationship with osteone diameter (B22) serve as an objective prediction of bone age. The importance of these features was revealed using random forest during EDA, since the correlation coefficients of the characteristics obtained through their analysis by descriptive statistics were 0.4 and 0.3, respectively. The circumstance proves efficient usage of random forest for the stated objective. Quantitative estimation of osseous tissue changing processes is extensively used as an objective measure of the biological age. While a person grows, develops, and ages, the evidences of an increasing number of structure element cycles are accumulating, therefore, the number of osteones with restricted central part (В18) is also consistently an attributive age index. The importance of age-related changes of spongy substance of the lower epiphysisа was noted: the number of osteones (В13), dimensional characteristics of the cartilaginous tissue area (В7).

**Figure 3:**
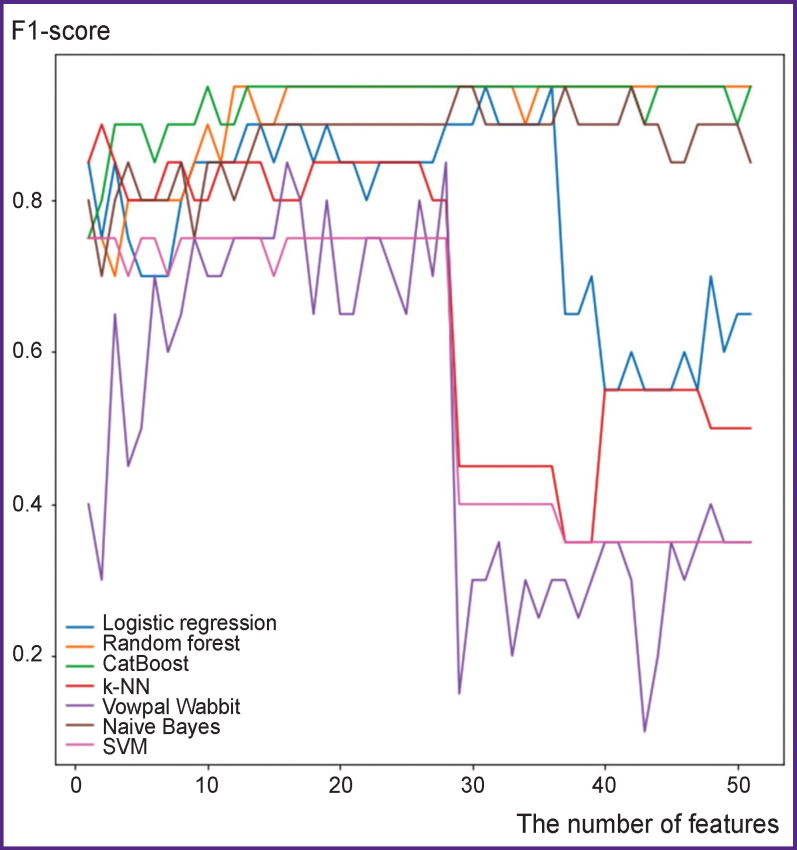
Dependence of F1-score value on the number of features used

The study analyzed the dependence of classifiers’ work quality on the number of features sorted out by importance decrease. It should be noted that for the algorithms based on decision trees technique (CatBoost, random forest) and applied for two-dimensional displays aimed at further imaging, the quality remains nearly the same up to using all initial features; that can be explained by the fact that these algorithms may select features themselves. Other algorithms are found to have the tendency for quality degradation after using more than 28 features. For this reason, exactly 28 features were selected (Figure 3).

**Figure 4:**
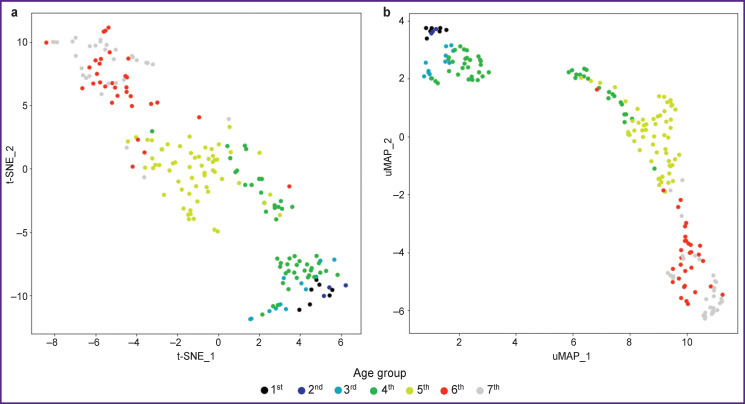
Two-dimensional display of initial data after feature selection: (а) using t-SNE; (b) using uMAP

After feature selection, the data were re-imaged (Figure 4). The more noticeable cluster structure was revealed. Moreover, neighboring age groups are closely located, while persons with a greater age gap are far from each other. t-SNE and uMAP techniques cannot handle missing data, so the missing values in initial data were filled by the feature value in the previous person in age-sorted-out data. It could make some points get into neighboring clusters, but even considering this fact, there were no situations when a person got into a cluster, the average age of which was very much different from his own.

To solve the problem, we compared the operational efficiency of classifiers. Table 2 demonstrates the results.

**Table 2:** Operating quality of algorithms on studied data

Algorithm name	F1-score
k-NN	0.83
SGD	0.85
SVM	0.85
Logistic regression	0.86
Naive Bayes	0.89
CatBoost	0.90
Random forest	0.91

**Figure 5:**
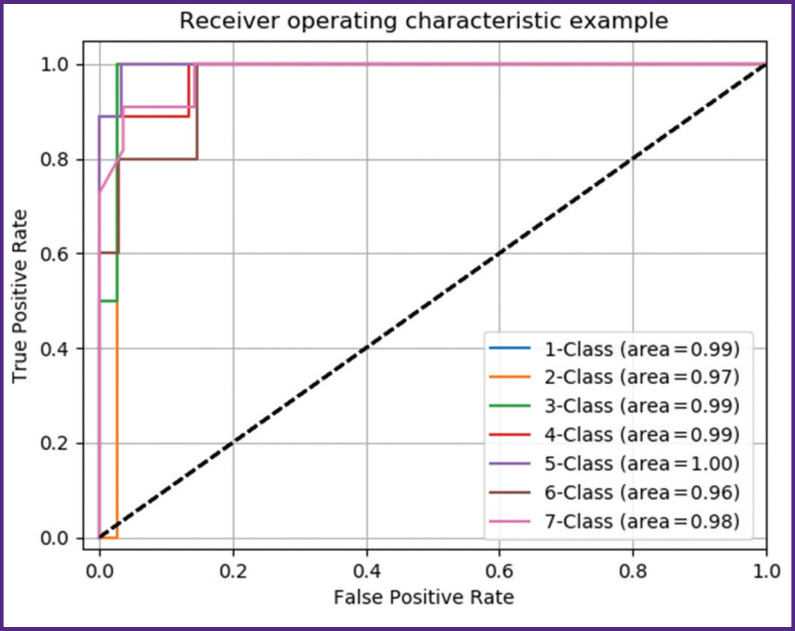
ROC curves for each age group

For further experiments, we chose random forest algorithm, since it showed the best performance on the data considered. The model quality was evaluated using cross-validation on 5 iterations. Confusion matrix (representation of real and predicted by algorithms class marks) and ROC curves for each class particularly were plotted (Figure 5). An error curve (receiver operating characteristic, ROC) is a curve, which enables to assess classification quality: it shows the relation between the proportion of objects from the total number of objects of positive classes, which were classified correctly (classification algorithm sensitivity), and the proportion of objects from the total number of negative class objects, which were referred to a positive class by mistake (classification algorithm specificity) when changing a decision rule threshold. The area under curve serves as a numerical characteristic of the model operating quality.

According to the data of the error matrix and ROC curves, the assessment of classification accuracy of objects by the developed model, the objects belonging to the 1st, 2nd, 3rd, 6th age groups, is 100%; those belonging to group 4 — 90%; group 5 — 92%. Steady fragmentation of these groups is the reflection of fundamental processes of postnatal ontogenesis in the chondro-osseous system. A maturation stage in groups 1–3 is changed by stabilization (group 4) and is ended by involutive transformations (groups 5–7). The least accuracy was found in age group 7. In people over 75, the condition of tissues and organs is due to both: age involution and also the resulting effect of the bulk of attending factors (diseases, medication intake, bad habits, nutrition, lifestyle, etc.), which have a marked cumulative effect.

**Figure 6:**
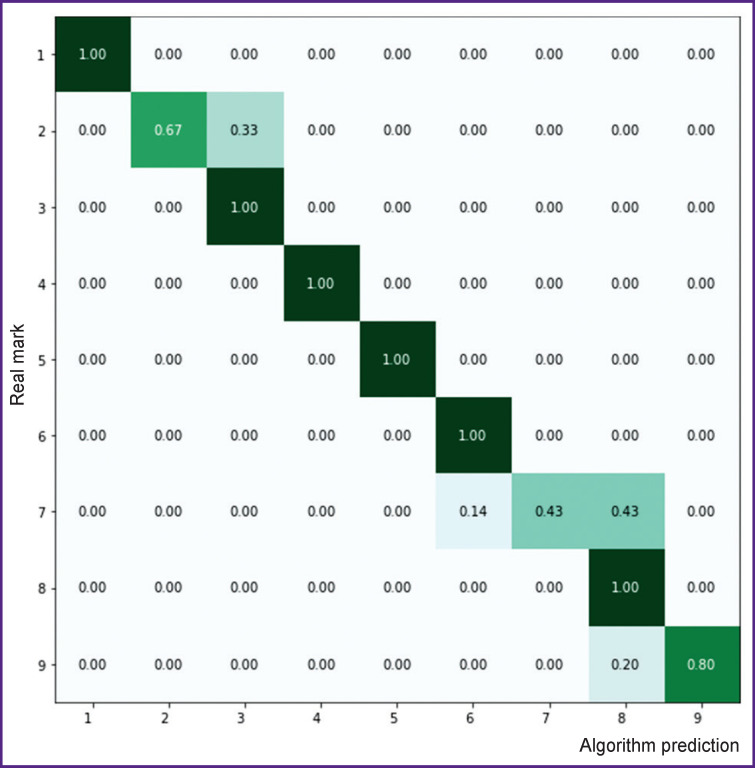
Error matrix

Figure 6 shows the result evaluations of the classification by the groups corresponding to a ten-year interval. Classification accuracy of group 2 (11–20 years) is 67%. The interval includes the periods of active growth and development of all organs and systems, puberty period that can result in uneven and heterogeneous indices. Therefore, relatively low accuracy is related to an age range of this age group. Classification accuracy decrease in group 7 (61–70 years), on the one hand, is related to the effect associated with the age of diseases, and on the other hand — it is in this decade when we found some retardation of age involution. These circumstances, to our opinion, can explain significant data scattering, and as a direct consequence, the objects could fall within neighboring groups. According to the error matrix data, classification accuracy of the 1st, 3rd, 4th, 5th, 6th, 8th age intervals on validation sampling is 100%, and for the 9th interval — 80%.

## Discussion

Histomorphometry of osseous tissue has been used for a long time to determine age [19–21]. There are many different modifications of techniques based on quantitative accounting of rearranging processes in osseous and cartilaginous tissues, as well as specimens (their selection) for investigations [22– 24]. We are in agreement with the authors of the study [24] about the necessity to refuse the statement that osseous tissue remodeling occurs at unpredictable speed. In its turn, it means the refusal to use linear models and causes further development of histomorphometric methods.

The analysis of histological quantitative variables demonstrates that they exhibit complex relations with age. Their relationship with gender, health condition (the presence of diseases, medication intake), biomechanics is no less important. It should prevent “simple” models creation — equations of linear regression — using universal indices of histological age estimation. That is why in our research to study a complex of histomorphometric indices we used the algorithms, which enables to analyze nonlinear dependencies (for instance, SVM with pertinent kernel, decision tree) and the techniques based on them, such as random forest or CatBoost. For imaging similar data, t-SNE and uMAP are expected to work better than classical methods, such as principal component analysis (PCA), which is used for similar purposes and demonstrates the notably less informative result [15]. The classification accuracy (±30 years) achieved by the authors of the work [15] can be related to the fact that there were studied the databases of qualitative estimation of morphological changes in pubic articulation.

A histomorphometric method, the findings of which were the material for the present study, is a quantitative measure and enables impartially approach age diagnostics. The obtained results showed feature space reduction to be a requisite measure resulting in no loss of classification quality. The operating efficiency of algorithms depends on the number of features, and in case there are 20–30 features, an adequate accuracy is attained. Then, it decreases up to the accuracy of linear classifiers. It should be emphasized that it is referred to the aggregate features. Maximum accuracy and reliability of the ultimate result were achieved at an integrated assessment of age-related changes of different types of osseous and cartilaginous tissues.

The application of an advanced technique of nonlinear dimensional reduction uMAP combined with a well-reputed t-SNE after feature selection provided an opportunity to observe the manifestation in the data of cluster structure, which was absent at a primary stage. It goes to show, on the one hand, the practicability of feature filtering by their informative value, and on the other hand — the correctness of the selection made. The studies also demonstrated feature space reduction to be a requisite measure resulting in no classification quality loss. Classification of objects using a ranked list of histomorphometric indices enabled us to obtain significant results concerning diagnostic accuracy and reliability of the required age group. A decision tree technique demonstrated the capability to independently select features in the course of work. Regardless of their quantity, the technique gives preference to the most informative ones. Random forest algorithm appeared to be the most productive among other classifiers considered in the study to solve the assigned initial objective; such circumstance confirms the advantage of the applied algorithm to achieve the stated objective.

## Conclusion

The obtained results proved the prospectiveness of machine learning techniques used in forensic expert medical practice to determine age, since the techniques showed rather high (about 90%) accuracy of the end result.

The carried out study using data mining enabled to arrange an optimal set of informative histomorphometric features of age-related changes, which is reasonable to use in order to establish a digital database as a constructive basis for data accumulation and systematization in forensic age determination. The formation of such lists enables to unify further researches in age morphology, and thereby extend “training” array data for prognoses. In fact, the issues with a learning sample (a great number of various features in a small number of observations) are the main constraining factor when implementing machine learning techniques in medicine.

The study of age involution principles with the formation of data warehouse of quantitative characteristics (ageing biomarkers) is a fundamental scientific challenge. High social significance of such researches is due to an increasing proportion of elderly. The obtained results are likely to be of interest for different medical spheres and biology including personified medicine.
